# Mechanical force induces mitophagy-mediated anaerobic oxidation in periodontal ligament stem cells

**DOI:** 10.1186/s11658-023-00453-w

**Published:** 2023-07-21

**Authors:** Zijie Zhang, Shuyue Cui, Yajing Fu, Jixiao Wang, Jiani Liu, Fulan Wei

**Affiliations:** Department of Orthodontics, School and Hospital of Stomatology, Cheeloo College of Medicine, Shandong University & Shandong Key Laboratory of Oral Tissue Regeneration & Shandong Engineering Laboratory for Dental Materials and Oral Tissue Regeneration & Shandong Provincial Clinical Research Center for Oral Diseases, Shandong University Cheeloo College of Medicine, No.44-1 Wenhua Road West, Jinan, 250012 Shandong China

**Keywords:** Mechanical force, Stem cells, Metabolism, Mitophagy

## Abstract

**Background:**

The preference for glucose oxidative mode has crucial impacts on various physiological activities, including determining stem cell fate. External mechanical factors can play a decisive role in regulating critical metabolic enzymes and pathways of stem cells. Periodontal ligament stem cells (PDLSCs) are momentous effector cells that transform mechanical force into biological signals during the reconstruction of alveolar bone. However, mechanical stimuli-induced alteration of oxidative characteristics in PDLSCs and the underlying mechanisms have not been fully elucidated.

**Methods:**

Herein, we examined the expression of LDH and COX4 by qRT-PCR, western blot, immunohistochemistry and immunofluorescence. We detected metabolites of lactic acid and reactive oxygen species for functional tests. We used tetramethylrhodamine methyl ester (TMRM) staining and a transmission electron microscope to clarify the mitochondrial status. After using western blot and immunofluorescence to clarify the change of DRP1, we further examined MFF, PINK1, and PARKIN by western blot. We used cyclosporin A (CsA) to confirm the regulation of mitophagy and ceased the stretching as a rescue experiment.

**Results:**

Herein, we ascertained that mechanical force could increase the level of LDH and decrease the expression of COX4 in PDLSCs. Simultaneously, the yield of reactive oxygen species (ROS) in PDLSC reduced after stretching, while lactate acid augmented significantly. Furthermore, mitochondrial function in PDLSCs was negatively affected by impaired mitochondrial membrane potential (MMP) under mechanical force, and the augment of mitochondrial fission further induced PRKN-dependent mitophagy, which was confirmed by the rescue experiments via blocking mitophagy. As a reversible physiological stimulation, the anaerobic preference of PDLSCs altered by mechanical force could restore after the cessation of force stimulation.

**Conclusions:**

Altogether, our study demonstrates that PDLSCs under mechanical force preferred anaerobic oxidation induced by the affected mitochondrial dynamics, especially mitophagy. Our findings support an association between mechanical stimulation and the oxidative profile of stem cells, which may shed light on the mechanical guidance of stem cell maintenance and commitment, and lay a molecular foundation for periodontal tissue regeneration.

**Graphical Abstract:**

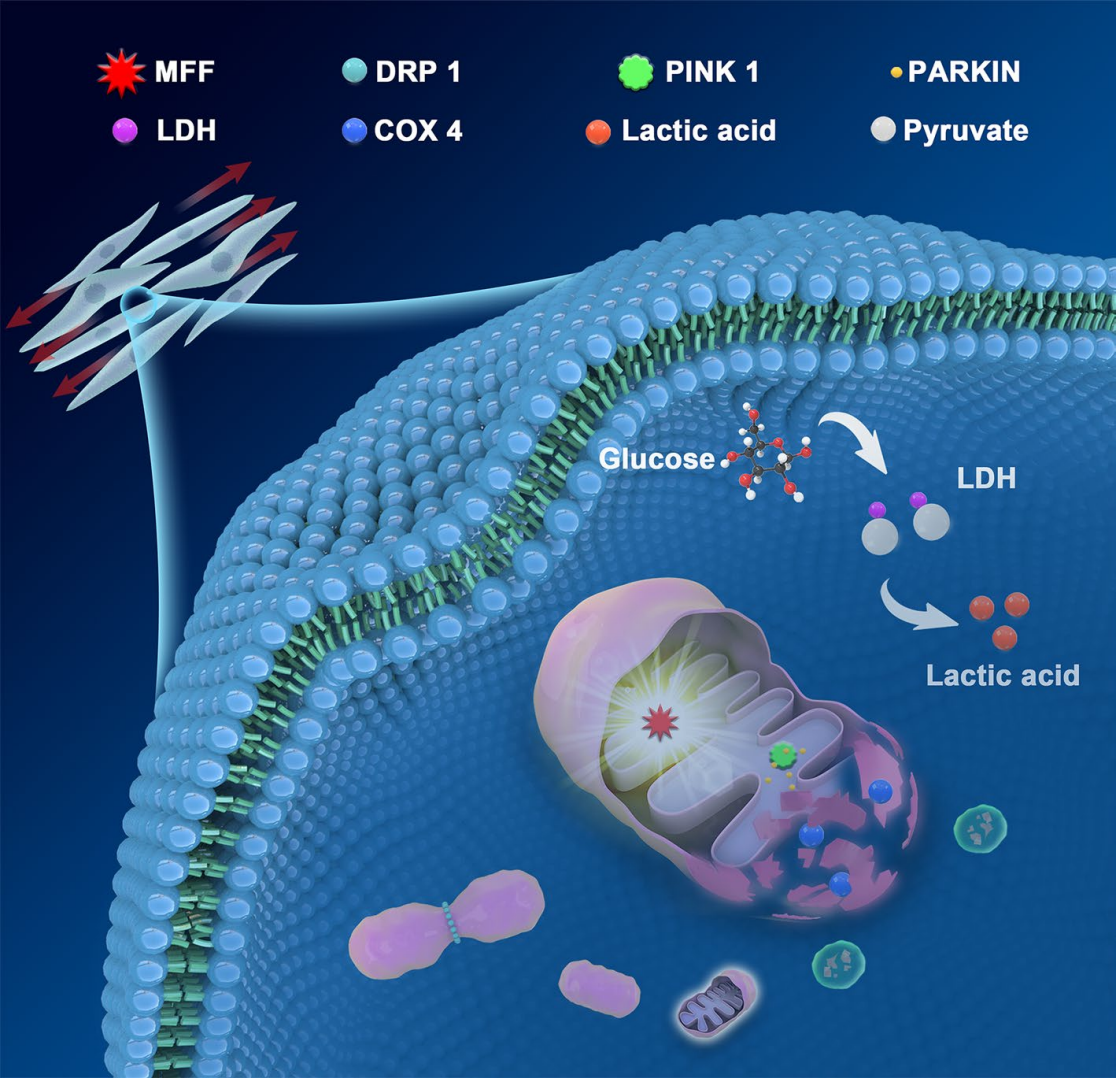

**Supplementary Information:**

The online version contains supplementary material available at 10.1186/s11658-023-00453-w.

## Background

The mechanical cues play a pivotal role in various physiological processes [1, 2] and even contribute to determining the fate of uncommitted stem cells [3]. As a type of target cells sensitive to mechanical stress, periodontal ligament stem cells (PDLSCs) can respond to mechanical stimulation to regulate multiple physiological processes, including proliferation and differentiation by fine-tuning the level of coding and non-coding genes [4, 5], signal pathways [6], and ion channels [7]. In addition, PDLSCs not only play a unique role as seed cells in bone reconstruction and regeneration of periodontal tissue, but also show extraordinary therapeutic potential in the regeneration of other homologous tissues [8, 9]. Therefore, it is essential to elucidate the adaptive changes and self-renewal capacity of PDLSCs under mechanical stimuli.

The physiological behavior of pluripotency stem cells (PSCs) under mechanical force is affected by various factors, such as gene and protein expression, signal pathway regulation, and metabolic key enzymes and processes [4, 7, 10]. Considerable studies have clarified that metabolic factors, especially the preference for glucose oxidation mode, have a crucial impact on the determination of stem cell fate, prevention of cellular stress, and maintenance of pluripotency [11, 12]. Compared with the classic regulation of aerobic and anaerobic oxidation based on oxygen supply, the oxidative homeostasis of PSCs with high plasticity tends to anaerobic oxidation to adapt to stem cell maintenance and commitment [12]. The spontaneous regulation of glucose oxidation mainly depends on mitochondria status, which refers to mitochondrial dynamics [13], including mitochondrial fusion, fission, and mitophagy [14, 15]. Additionally, several studies in the stomatology field have found that mechanical forces affect the profile of metabolic enzymes in gingival crevicular fluid (GCF) [16, 17]. However, mechanically-induced alteration of oxidative characteristics in PDLSCs and the underlying mechanisms have not been fully elucidated.

To address this knowledge gap, this study aimed to illuminate the adaptive alteration of oxidative profile in PDLSCs under mechanical stimuli. We found that the oxidation mode of mechanically affected PDLSCs inclines to anaerobic oxidation via augmented mitophagy. This regulation of oxidative preference can reverse after the removal of force-loading to avoid affecting the differentiation potential of PDLSC as a stem cell. These findings could provide a promising avenue for mechanical-regulating oxidative preference and the subsequent potential of pluripotency and stem cell commitment, which is not confined to tissue regeneration in stomatology, but also of great significance for craniomaxillofacial and embryonic development.

## Methods

### Cell culture and identification

Human PDLSCs were isolated from the periodontal tissues of premolars extracted from the Department of Oral Maxillofacial Surgery, School of Stomatology, Shandong University (Jinan, China). Primary PDLSCs were obtained from periodontal tissues which were cut into 2 mm^2^ pieces. PDLSCs were incubated at 37 °C in a humidified atmosphere containing 5% CO_2_ in MEM-alpha (Biological Industries, Israel) with 10% fetal bovine serum (FBS, Lonsera, China) at 37 °C in a humidified atmosphere with 5% CO_2_. Selected surface markers of the PDLSCs, including CD31, CD34, CD45, CD90, CD146, and Stro-1, were analyzed by flow cytometry.

### Animal model

A total of 18 six-week-old male Wistar rats (Charles River, USA) with an average weight of approximately 200 g were kept for 3 days under a photocycle alternating between 12 h of day and 12 h of night, with no limitation to obtain food and water. The left maxillary first molar of each rat was ligatured with the upper incisor on the same side via a nickel–titanium closed-coil spring, tied to the teeth by a 0.25 mm thread of stainless steel (TOMY, Japan). The thread was secured with photocurable resin in a groove drilled on the surface of the upper incisors. The entire dental device provided approximately 20 g of force. The rats were then equally divided into six groups, designated as A, B, C, D, E, and F, for which the dental device was maintained in place for the 0, 1, 3, 7, 14, and 21 days, respectively. Each group contains three independent samples for statistical analysis. In the modeling process, the order of operation on each rat and the position of the cage were random. Following over injection of 3% pentobarbital sodium, the left dentition of each rat as tooth movement model and right dentition as control, together with its alveolar bone, was harvested and fixed in 10% formalin for 24 h. The bone tissue was then decalcified in 14% ethylenediaminetetraacetic acid (EDTA) for 3 months. The paraffin-embedded tissue was cut into 5-μm thick sections, followed by alcohol gradient dehydration.

### Pluripotency assessment

The pluripotency of the PDLSCs was verified by evaluating their ability to undergo osteogenic, adipogenic, and chondrogenic differentiation. To induce osteogenesis, the PDLSCs were incubated with 50 mg/L ascorbic acid, 5 mM β‐glycerophosphate, and 10 nM dexamethasone in MEM-alpha with 10% FBS for 21 days. The induced PDLSCs were then fixed with 4% paraformaldehyde for 10 min at room temperature before being stained with 1% alizarin red (Solarbio, China).

To induce adipogenesis, the PDLSCs were incubated with 60 mM indomethacin, 10 mg/L insulin, 0.5 mM IBMX, and 0.5 μM dexamethasone, in MEM-alpha with 10% FBS for 28 days. The resultant cell population was fixed in 4% paraformaldehyde for 10 min before being stained with oil red O (Solarbio, China).

Chondrogenic differentiation of the PDLSCs was induced by an Ori Cell Bone Marrow Mesenchymal Stem Cell Chondrogenic Differentiation Kit (Cyagen, China) according to the manufacturer’s instructions. The PDLSCs were cultured in the Chondrogenic Differentiation Basal Medium for 28 days, and the resultant cartilaginous mass was fixed in 10% formalin for 24 h and then embedded in paraffin. The cartilage sections were stained by alcian blue (Cyagen, China).

### Application of mechanical force in vitro

The PDLSCs prior to the fifth passage were seeded onto collagen I-coated, amino silicone-bottomed 6-well cell culture plates (Flexcell International, USA) at a density of 2.0 × 10^5^ PDLSCs per well. Cells were cultured under the standard conditions described above. After reaching ~ 80% confluence, PDLSCs were subjected to serum deprivation at 2% FBS (Lonsera, China) for 24 h. The cells were then mechanically stretched with the FX-6000 Tension System (Flexcell International, USA) at 10% elongation and 0.5 Hz. The control group of cells was cultured under the identical condition as above, but without applying the mechanical stimuli.

### RNA extraction and quantitative reverse-transcription polymerase chain reaction (qRT-PCR)

Total RNA was isolated by RNAiso Plus (Takara, Japan) following the manufacturer’s instructions. The cDNA reverse-transcription was using the PrimeScript RT Reagent Kit with gDNA Eraser (Takara, Japan). Subsequently, qRT-PCR was performed in triplicate on the LightCycler 480 system (Roche Diagnostics, Switzerland) with TB Green Premix Ex Taq II (Takara, Japan). Gene expression levels were quantified on the basis of the 2^−ΔCt^ method. All qRT-PCR primer sequences used in this study are listed in Table 1.

**Table 1:** The primers and antibodies used in this study

Primers	
LDHA	Forward: TTGTTGGGGTTGGTGCTGTTG
	Reverse: AAGAGCAAGTTCATCTGCCAAG
COX4	Forward: CGATCACCTTGACGGACGAG
	Reverse: TCTTCTCATAGTCCCAGCGC
Antibodies for western blot	
LDH	Abcam, anti-lactate dehydrogenase antibody, ab52488
COX4	CST, COX IV (3E11) rabbit mAb, #4850
DRP1	Proteintech, DRP1 polyclonal antibody, 12957-1-AP
MFN2	Proteintech, MFN2 polyclonal antibody, 12186-1-AP
MFF	Abcam, anti-MFF antibody, ab129075
PINK1	Proteintech, PINK1 polyclonal antibody, 23274-1-AP
PARKIN	Proteintech, PARK2/Parkin monoclonal antibody, 66674-1-Ig
GAPDH	Proteintech, GAPDH polyclonal antibody, 10494-1-AP
β-actin	Proteintech, beta actin polyclonal antibody, 20536-1-AP
Antibodies for immunofluorescence staining	
LDH	Abcam, anti-lactate dehydrogenase antibody, ab52488
COX4	CST, COX IV (3E11) rabbit mAb, #4850
ldh	Abcam, anti-lactate dehydrogenase antibody, ab52488
Cox4	CST, COX IV (3E11) rabbit mAb, #4850
DRP1	Proteintech, DRP1 (C-terminal) polyclonal antibody, 12957-1-AP
VDAC1	Proteintech, VDAC1/Porin monoclonal antibody, 66345-1-Ig
Drp1	Proteintech, DRP1 (C-terminal) polyclonal antibody, 12957-1-AP
Vdac1	Proteintech, VDAC1/Porin monoclonal antibody, 66345-1-Ig
Antibodies for immunohistochemical staining	
ldh	Abcam, anti-lactate dehydrogenase antibody, ab52488
cox4	CST, COX IV (3E11) rabbit mAb, #4850

### Western blot

The PDLSCs washed with prechilled phosphate-buffered saline (PBS) were lysed in a radioimmunoprecipitation assay buffer (Solarbio Science, China) containing 1% phenylmethylsulfonyl fluoride (Solarbio Science, China) for 5 min at 4 °C, followed by three cycles of 5 s sonication on ice. The cell lysate was centrifuged at 12,000 rpm at 4 °C for 15 min to obtain the protein lysate. The proteins were denatured, separated in a 10% sodium dodecyl sulfate–polyacrylamide gel electrophoresis (SDS-PAGE) gel, and transferred to a 0.2-μm polyvinylidene fluoride membrane (Millipore, USA). The membrane was blocked with 5% skimmed milk in Tris-buffered saline containing 0.1% Tween-20 (TBS-T) at pH 7.2, then incubated overnight at 4 °C with primary antibodies of choice. After washing three times with TBS-T, the membrane was incubated for 1 h with horseradish peroxidase (HRP)-conjugated secondary antibodies of choice at room temperature and washed again with TBS-T. Protein bands were visualized by an Amersham Imager 600 (General Electric, USA) with ECL Western Blotting Substrate (Biosharp, China). Densitometric quantification of the protein bands was conducted by using ImageJ (National Institutes of Health, USA). All primary and secondary antibodies used for western blot in this study are listed in Table 1.

### Immunofluorescence

The PDLSCs fixed with 4% paraformaldehyde were permeabilized with 0.1% Triton X-100 in PBS for 20 min, rinsed with TBS, and blocked with PBS contained 5% bovine serum albumin (BSA, Sigma-Aldrich, USA) for 1 h. The cells were incubated with primary antibodies of choice at 4 °C overnight and washed three times with PBS, followed by staining first with fluorophore-conjugated secondary antibodies of choice for 1 h at room temperature and then with 4′,6-diamidino-2-phenylindole (DAPI, Solarbio, China) for 5 min. The results were visualized under an IX73 inverted microscope (Olympus, Japan). Immunofluorescence characterization of the murine alveolar bone tissues followed the same procedures, except that the sections were incubated with 0.1% trypsin (Solarbio, China) at 37 °C for 20 min for antigen retrieval prior to blocking. The stained tissues were visualized under a BX51 fluorescence microscope (Olympus, Japan) and the LSM880 confocal laser scanning microscope (Carl Zeiss, Germany). Densitometric quantification of the fluorescence intensity was conducted by using ImageJ (National Institutes of Health, USA).

All primary and secondary antibodies used for immunofluorescence characterizations in this study are listed in Table 1.

### Immunohistochemistry

The murine alveolar bone tissue sections were processed up to the step of antigen retrieval described above and then incubated with 3% hydrogen peroxide at 37 °C for 20 min. The samples blocked with BSA were then incubated with primary antibodies and subsequent HRP-conjugated secondary antibodies following the same procedures mentioned earlier. Subsequently, the tissue sections were stained with diaminobenzidine and hematoxylin (Solarbio, China), then visualized by a BX51 fluorescence microscope. Densitometric quantification of staining positive cells was conducted by using ImageJ (National Institutes of Health, USA). All primary and secondary antibodies used for immunohistochemical staining in this study are listed in Table 1.

### Measurement of lactic acid production

Production of lactic acid in the PDLSCs was measured with the Lactic Acid Assay Kit (Nanjing Jiancheng Bioengineering, China) following the manufacturer’s protocol. Briefly, 0.02 mL of the culture supernatant was incubated with 1 mL of the enzyme working solution and 0.2 mL of the chromogenic solution at 37 °C for 10 min, followed by adding 2 mL of the Stop solution. Lactic acid concentrations were detected at 530 nm on a SPECTROstar Nano Microplate Reader (BMG Labtech, Germany).

### Detection of reactive oxygen species

Total ROS in the PDLSCs was measured with the Reactive Oxygen Species (ROS) Colorimetric Assay Kit (Elabscience, China) following the manufacturer’s protocol. Briefly, the PDLSCs were incubated with the buffer solution containing 10 mM 2’,7’-dichlorofluorescein diacetate (DCFH-DA) at 37 °C for 30 min, washed with the buffer solution and then examined by both visual observations, under an IX73 inverted microscope, and flow cytometry analysis.

### Measurement of mitochondrial membrane potential (MMP)

The MMP of the PDLSCs was measured with the MitoProbe TMRM Assay Kit (Thermo Fisher Scientific, USA) according to the manufacturer’s protocol. In brief, the cells were treated with the cell staining solution for 30 min at 37 °C, and then visualized under an IX73 inverted microscope, after being washed with PBS.

### Transmission electron microscopy (TEM)

The PDLSCs were fixed with 2.5% glutaraldehyde (Sigma Aldrich, USA) for 3 h at room temperature and then washed with PBS at pH 7.4. The PDLSCs were then fixed in 1% osmic acid for 1.5 h in the dark, followed by gradient dehydration in 50%, 70%, 80%, 90% ethanol, 90% acetone, and pure acetone, in sequence. After penetration with acetone and resin, PDLSCs were embedded with resin before sectioning with a thickness of 70 nm. BAF and DMSO were added 4 h in advance before fixation. The ultrastructure of cells was visualized by transmission electron microscope (HT-7800, Hitachi, Japan).

### Statistical analysis

All experimental data that were normally distributed were expressed as mean ± standard deviation from at least three independent experiments and analyzed using SPSS 19.0 (SPSS Inc., USA). Differences between two experimental groups were analyzed using the Student’s *t*-test, while differences between three groups were analyzed using one-way analysis of variance (ANOVA). *P < *0.05 was considered statistically significant.

## Results

### Biological characteristics identification of PDLSCs

**Fig. 1:**
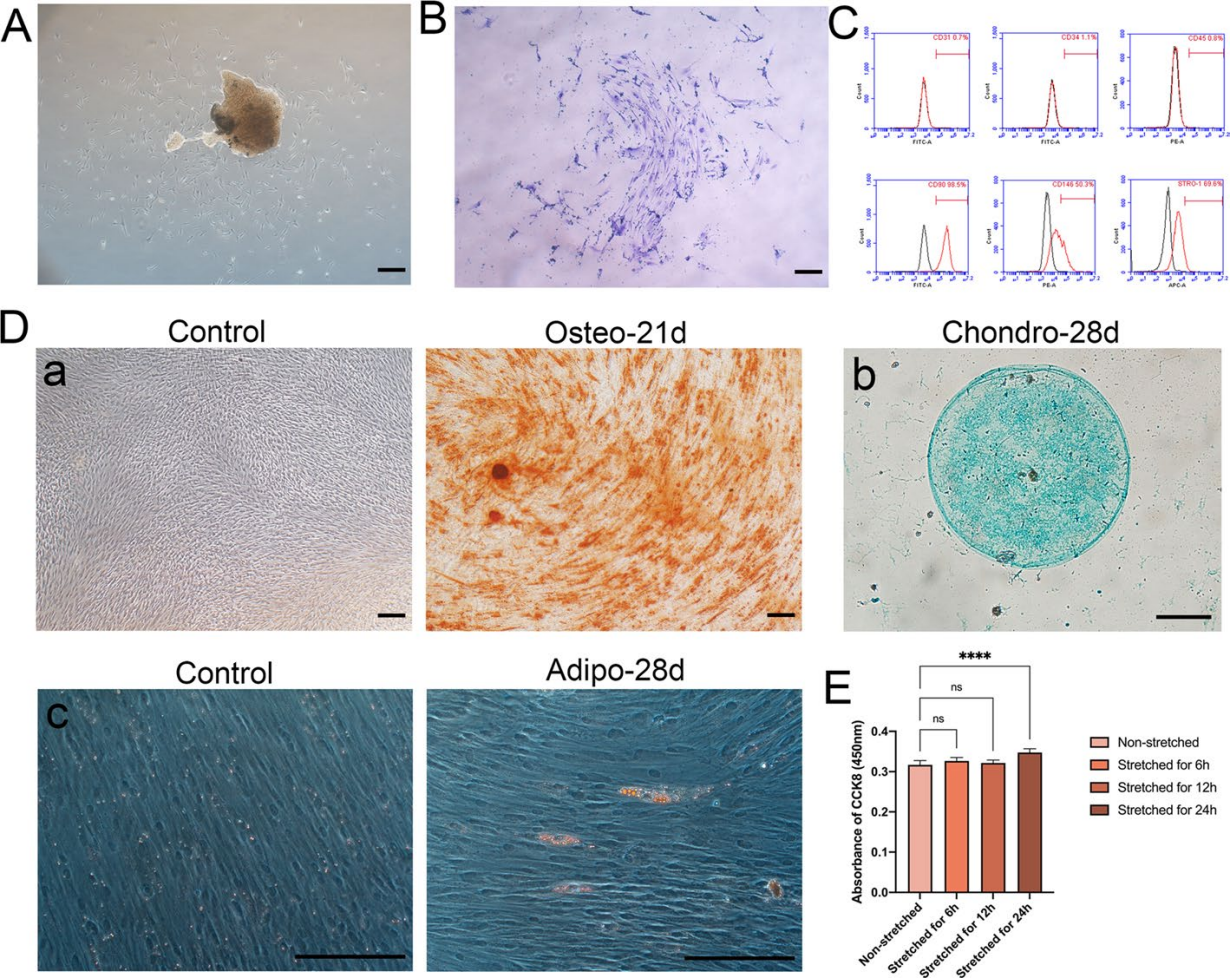
Biological characteristics identification of PDLSCs. **A** Cell morphology of primary periodontal ligament stem cells (PDLSCs). Scale bar = 200 μm. **B** Colony-forming assay stained by crystal violet. Scale bar = 200 μm. **C** Expression of cell surface markers CD31, CD34, CD45, CD90, CD146, and Stro-1 by flow cytometry. **D** (a) The alizarin red staining of PDLSCs after osteogenic induction for 0 days and 21 days. Scale bar = 200 μm. (b) The alcian blue staining of PDLSCs after cartilage induction for 28 days. Scale bar = 200 μm. (c) Oil red O staining of PDLSCs after adipogenic induction for 0 days and 28 days. Scale bar = 200 μm. **E** The CCK8 results of PDLSCs after stretching for 0, 6, 12, and 24 h. ****P < 0.0001

We first verified the biological characteristics to lay a proper cellular foundation for subsequent experiments. The cells harvested from cultured periodontal ligament were shown to be spindle-shaped under a light microscope (Fig. 1A) and possessed excellent proliferating capabilities based on the results of colony formation assay with crystal violet (Fig. 1B). Flow cytometric analysis demonstrated the expression of CD90, CD146, and Stro-1, as well as the absence of CD31, CD34, and CD45, in consistence with the properties of mesenchymal stem cells (Fig. 1C). The pluripotency of the cells was corroborated by their ability to undergo osteogenesis, chondrogenesis, and adipogenesis (Fig. 1D). For the mechanical side effect on cells, we conducted CCK8 assay and found that mechanical force did not affect the growth activity of PDLSCs. Additionally, the PDLSC activity was significantly enhanced after 24 h of force application (Fig. 1E). Collectively, these results confirmed our success in obtaining PDLSCs with multilineage differentiation properties from the periodontal ligament tissues.

### Mechanical stretching altered oxidative pattern in PDLSCs

**Fig. 2:**
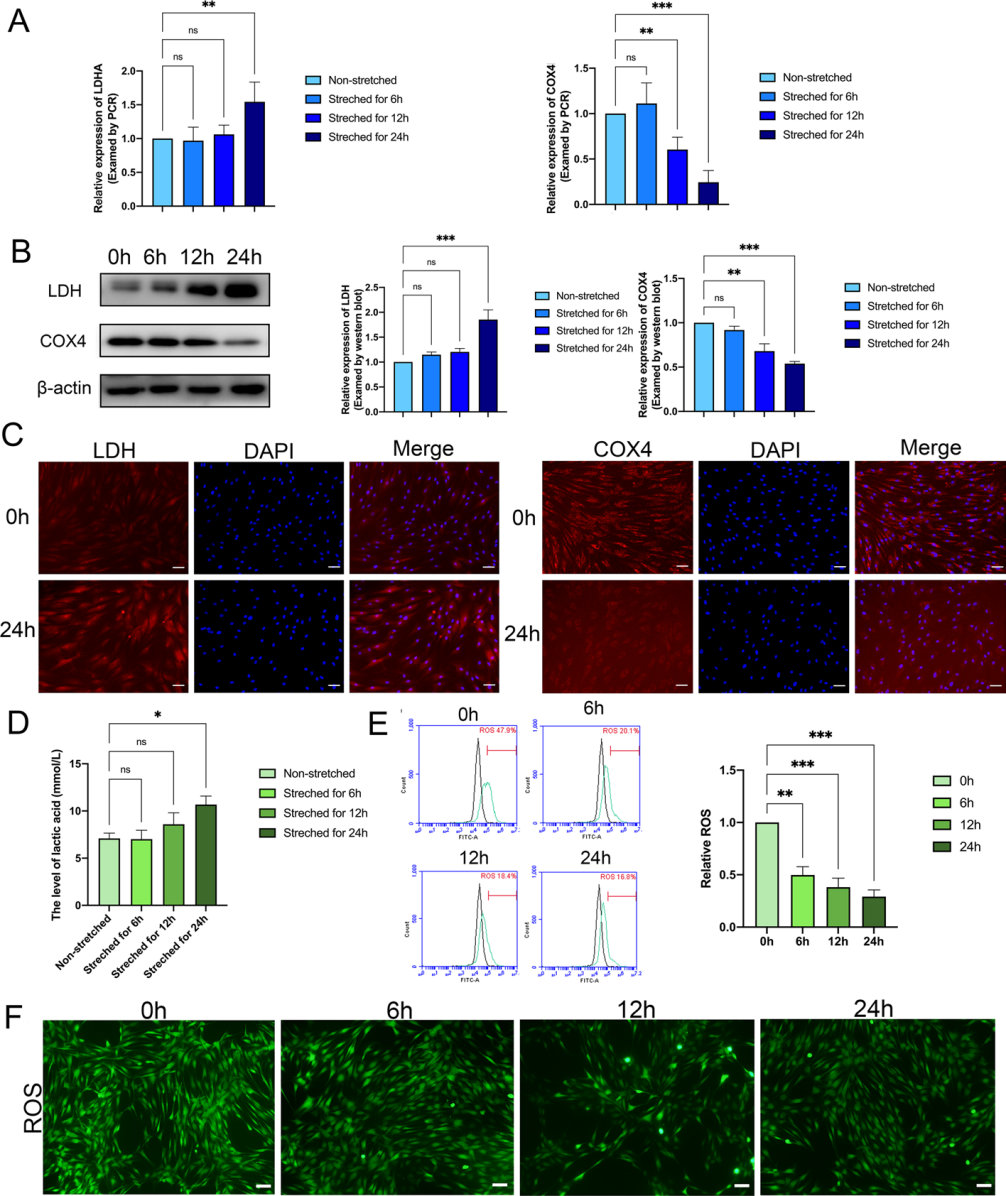
Mechanical stretching altered oxidative pattern in PDLSCs. **A** The relative RNA expression of LDH and COX4 in PDLSCs after stretching for 0, 6, 12, and 24 h. **B** The protein level of LDH and COX4 in PDLSCs after stretching for 0, 6, 12, and 24 h. **C** The expression of LDH and COX4 in PDLSCs stretched for 0 h and 24 h visualized by immunofluorescence staining. Scale bar = 50 μm. **D** The level of lactic acid produced by PDLSCs after stretching for 0, 6, 12, and 24 h. **E**, **F** The level of ROS in PDLSCs after 0, 6, 12, and 24 h of stretching. Scale bar = 100 μm. **P < 0.01, *P < 0.05, ***P < 0.001, *ns* no significance

To clarify the modification of the oxidative pattern, we analyzed the expression of selected oxidative genes and proteins in mechanically stretched PDLSCs. Since the difference between aerobic and anaerobic oxidation occurs after the common undergo of glycolysis, we chose cytochrome c oxidase subunit 4 (COX4), the terminal complex of the electron transport chain (ETC), as the representative biomarker of aerobic oxidation [18, 19], and lactate dehydrogenase (LDH), which is the last key enzyme before generating lactic acid as anaerobic oxidation factor [20]. As seen, both qRT-PCR and western blot studies revealed a notable enhancement in the expression of LDH, and a decline in that of COX4, in stretched PDLSCs at transcriptional and protein level (Fig. 2A, B). These findings were echoed by immunofluorescence staining of LDH and COX4 (Fig. 2C). Furthermore, the level of lactic acid increased remarkably in the PDLSCs stretched for 24 h compared with the control that was not under any mechanical stimuli (Fig. 2D). As an inevitable product of aerobic oxidation, reactive oxygen species (ROS) could reveal the level of aerobic oxidation in physiological states. The level of ROS performed a significant downward trend after mechanical stimuli in a time-dependent manner (Fig. 2E, F). In the light of these findings, we concluded that mechanical force could promote anaerobic oxidation and impair aerobic oxidation in PDLSCs.

### Application of mechanical force altered the expression of oxidation-related proteins in vivo

**Fig. 3:**
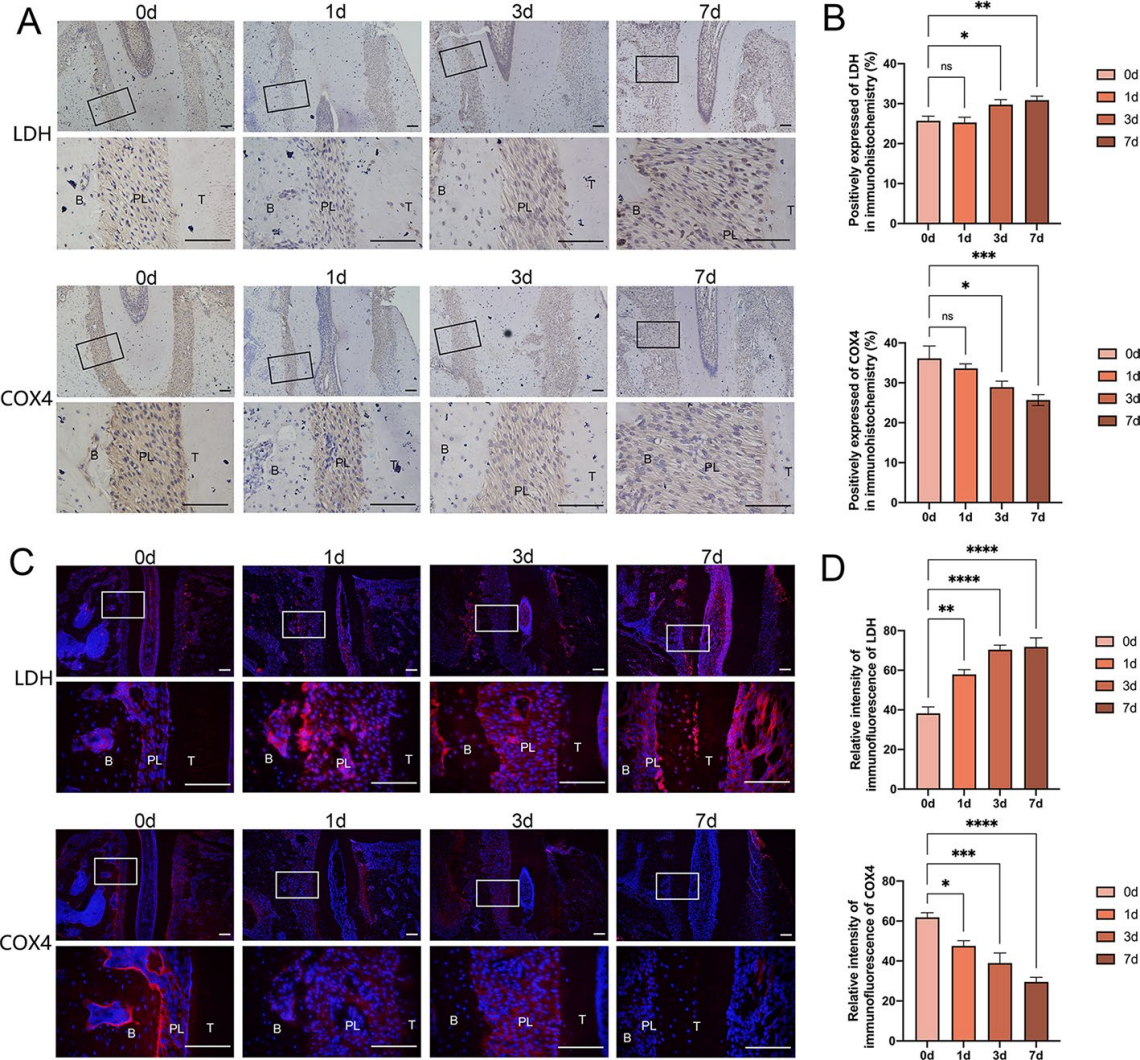
Application of mechanical force altered the expression of oxidation-related proteins in vivo. **A**, **B** The expression of LDH and COX4 in the periodontal ligament of Wistar rats after 0, 1, 3, and 7 days of tooth movement, visualized by immunohistochemical staining. Scale bar = 100 μm. **C**, **D** The expression of LDH and COX4 in the periodontal ligament of Wistar rats after 0, 1, 3, and 7 days of tooth movement visualized by immunofluorescence staining. Scale bar = 100 μm. B: alveolar bone, PL: periodontal ligament, T: tooth. *P < 0.05, **P < 0.01, ***P < 0.001, ****P < 0.0001. *ns* no significance

To explore whether the mechanical ordinance of the oxidative pattern we ascertained in PDLSCs could also be replicated in vivo, we measured the expression of LDH and COX4 in the periodontal ligament tissues during force-dominated tooth movement. On the basis of the immunohistochemical staining, the tissue level of LDH augmented significantly in the tooth movement of 3 and 7 days, compared with tooth movement with the device for only one day and the device-free control group (Fig. 3A, B). Conversely, the tissue level of COX4 showed a steady, significant decline with a longer duration of tooth movement (Fig. 3A, B). Consistent with the immunohistochemical results, immunofluorescence staining also provided clear evidence of elevated LDH expression and diminished COX4 levels in the mechanically stretched murine periodontal ligament tissues (Fig. 3C, D). As a result, experiment in vivo indicated a drop in aerobic oxidation and an elevation in anaerobic oxidation in periodontal tissues during mechanical-induced tooth movement, which was compatible with the in vitro experiment’s results.

### Mechanical force stimulated mitochondrial fission

**Fig. 4:**
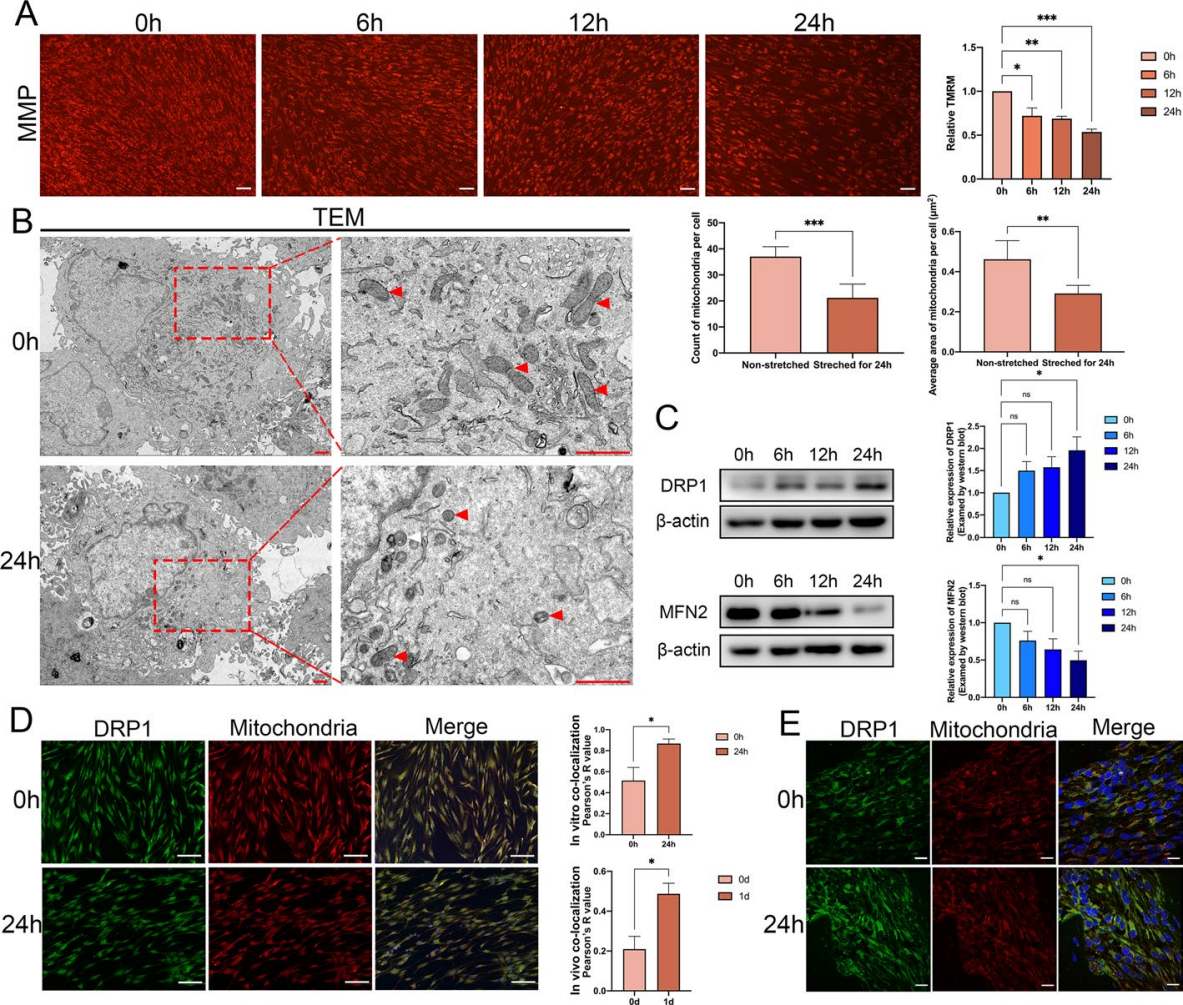
Mechanical force stimulated mitochondrial fission. **A** The MMP in PDLSCs after 0 h and 24 h of stretching, visualized by TMRM staining. Scale bar = 100 μm. **B** The morphology of mitochondria in PDLSCs after 0 h and 24 h of stretching visualized by TEM. The number and the average size of mitochondria in PDLSCs after 0 h and 24 h of stretching. Red arrow: mitochondria. Scale bar = 1 μm. **C** The protein level of DRP1 and MFN2 in PDLSCs after being stretched for 0, 6, 12, and 24 h. **D** The subcellular localization of DRP1 and mitochondria in PDLSCs stretched for 0 h and 24 h visualized by cell immunofluorescence staining. Scale bar = 100 μm. **E** The subcellular localization of DRP1 and mitochondria in murine periodontal ligament tissues stretched for 0 d and 1 d visualized by cell immunofluorescence staining. Scale bar = 10 μm. *P < 0.05, **P < 0.01, ***P < 0.001. *ns* no significance

Based on the results that lead to impairment of aerobic oxidation in PDLSCs under mechanical force and the close liaison between aerobic oxidation and mitochondria, we speculated that mechanical force might exhibit adverse effects on mitochondria. In support of this hypothesis, mitochondrial membrane potential (MMP) of PDLSCs displayed a decrease in fluorescence with the elapsed time of the mechanical stretching, which suggested the damage of mitochondria (Fig. 4A). As a clarification of how mitochondria were defective due to mechanical force, transmission electron microscopy (TEM) imaging illustrated that 24 h of mechanical stretching led to a dramatic drop in both the amount and average size of mitochondria in the PDLSCs (Fig. 4B), prompting us to speculate that there could be an imbalance between mitochondrial fusion and fission. We thus analyzed the expression of the representative markers, mitofusin-2 (MFN2) for mitochondrial fusion and dynamin-related protein 1 (DRP1) for fission in the PDLSCs that were mechanically stretched for different periods up to 24 h. In support of our assumption, the PDLSCs presented a steady ascent of DRP1 and abatement of MFN2 (Fig. 4C). We observed increased mitochondrial recruitment of DRP1 as a result of mechanical stimuli, based on subcellular localization studies via immunofluorescence microscopy (Fig. 4D). Similar results were derived from murine periodontal ligament tissues after 24 h of mechanical stretching (Fig. 4E). Taken together, the above results indicated that mechanical stretching could provoke mitochondrial fission.

### Mechanical force-led alteration of oxidation pattern in PDLSCs was induced by mitophagy

**Fig. 5:**
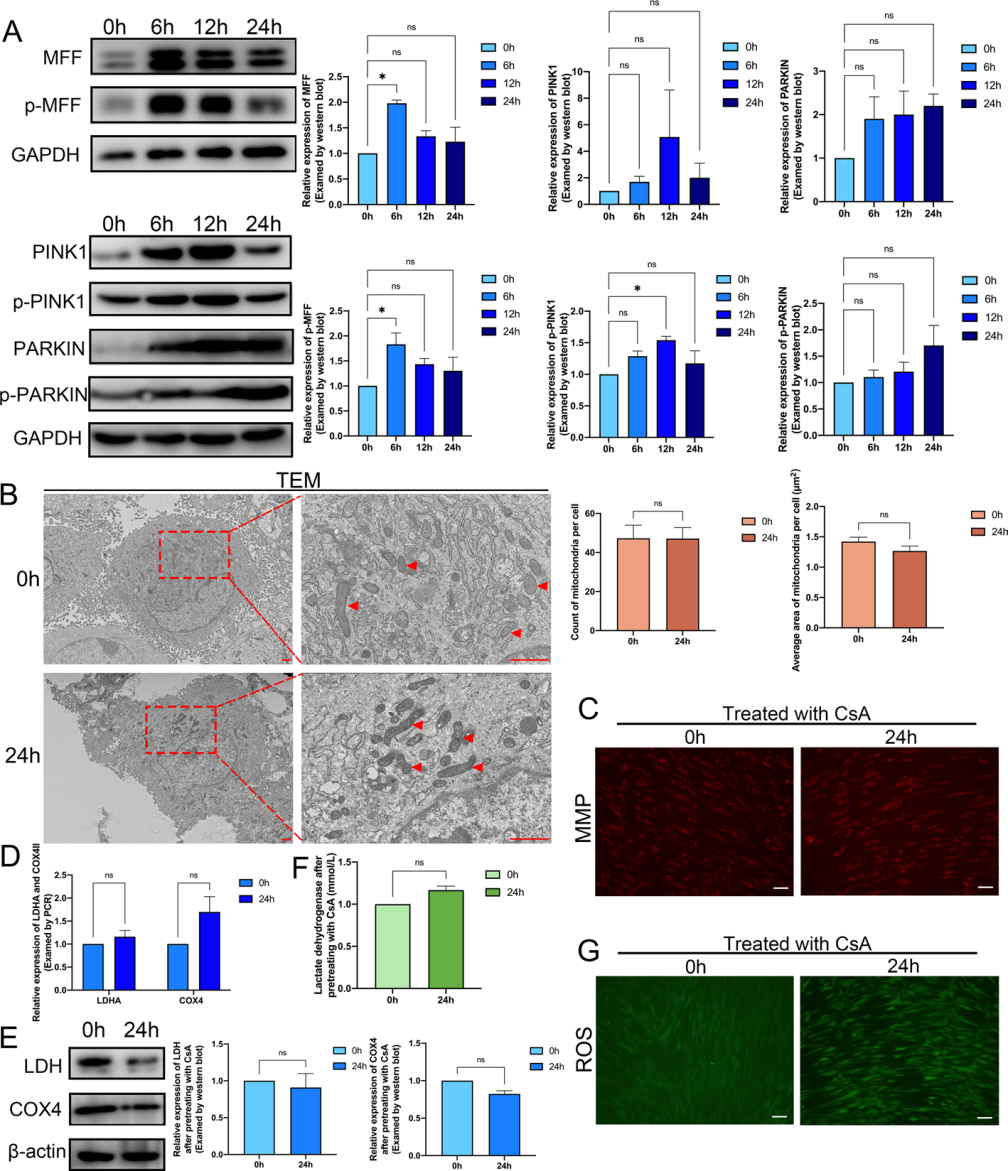
Mechanical force-led alteration of oxidation pattern in PDLSCs was induced by mitophagy. **A** The protein level of MFF, p-MFF, PINK1, p-PINK1, PARKIN, and p-PARKIN in PDLSCs after stretching for 0, 6, 12, and 24 h. **B** The morphology of mitochondria in CsA pretreated PDLSCs after 0 h and 24 h of stretching visualized by TEM. Red arrow: mitochondria. Scale bar = 1 μm. **C** The MMP in CsA pretreated PDLSCs after stretching for 0 h and 24 h visualized by TMRM staining. Scale bar = 100 μm. **D** The relative RNA expression of LDHA and COX4 in CsA pretreated PDLSCs after being stretched for 0 h and 24 h. **E** The protein level of LDH and COX4 in CsA pretreated PDLSCs after being stretched for 0 h and 24 h. **F** The level of lactic acid in CsA pretreated PDLSCs after stretching for 0 h and 24 h. **G** The level of ROS in CsA pretreated PDLSCs after stretching for 0 h and 24 h. Scale bar = 100 μm. *P < 0.05. *ns* no significance

As another decisive process of mitochondrial quality control, mitophagy showed a close liaison with mitochondrial fission [21]. After confirming the rising fission under mechanical force, we next assessed mitochondrial mitophagy in the mechanically stretched PDLSCs. We detected the expression of mitophagy indicator mitochondrial fission factor (MFF), which was also known to be a DRP1 receptor responsible for its translocation to mitochondria. We identified that the aggregate and phosphorylated levels of MFF were both significantly elevated by the mechanical stimuli in the PDLSCs, peaking at 6 h of continuous stretching (Fig. 5A). Previous reports suggested that PRKN-dependent mitophagy was closely associated with flawed MMPs [22, 23], so we examined PINK1 and PARKIN, the hallmark proteins of the PRKN-dependent pathway in PDLSCs under mechanical force. Concomitantly, we found that the total and phosphorylated levels of PINK1 and PARKIN increased after loading mechanical force, and the increased time was lagging compared with MFF (Fig. 5A). These results indicated that mitophagy was elevated in the early stage of stretching in PDLSCs. To inspect the effect of mitophagy on the oxidative pattern of PDLSCs under mechanical force, we pretreated PDLSCs with the mitophagy-specific inhibitor cyclosporin A (CsA) [26, 27]. The TEM images demonstrated that after CsA-pretreating, the stretched PDLSCs showed no significant discrepancy in either the amount and average size of mitochondria compared with non-stretched PDLSCs (Fig. 5B). TMRM staining also indicated that mitochondrial status represented by MMP was not impaired by stretching in CsA-pretreated PDLSCs (Fig. 5C). Additionally, the CsA-pretreated PDLSCs showed no substantial divergence in LDH and COX4 in the transcript and protein levels after mechanical force loading (Fig. 5D, E). Regarding metabolites, the production of lactic acid in CsA-pretreated PDLSCs did not change significantly compared with the non-stretched PDLSCs (Fig. 5F), and neither was ROS (Fig. 5G). The above results suggested that the oxidative pattern changes of PDLSCs after mechanical stimuli were caused by mitophagy.

### The oxidative pattern altered by mechanical force was restored after the cessation of mechanical force

**Fig. 6:**
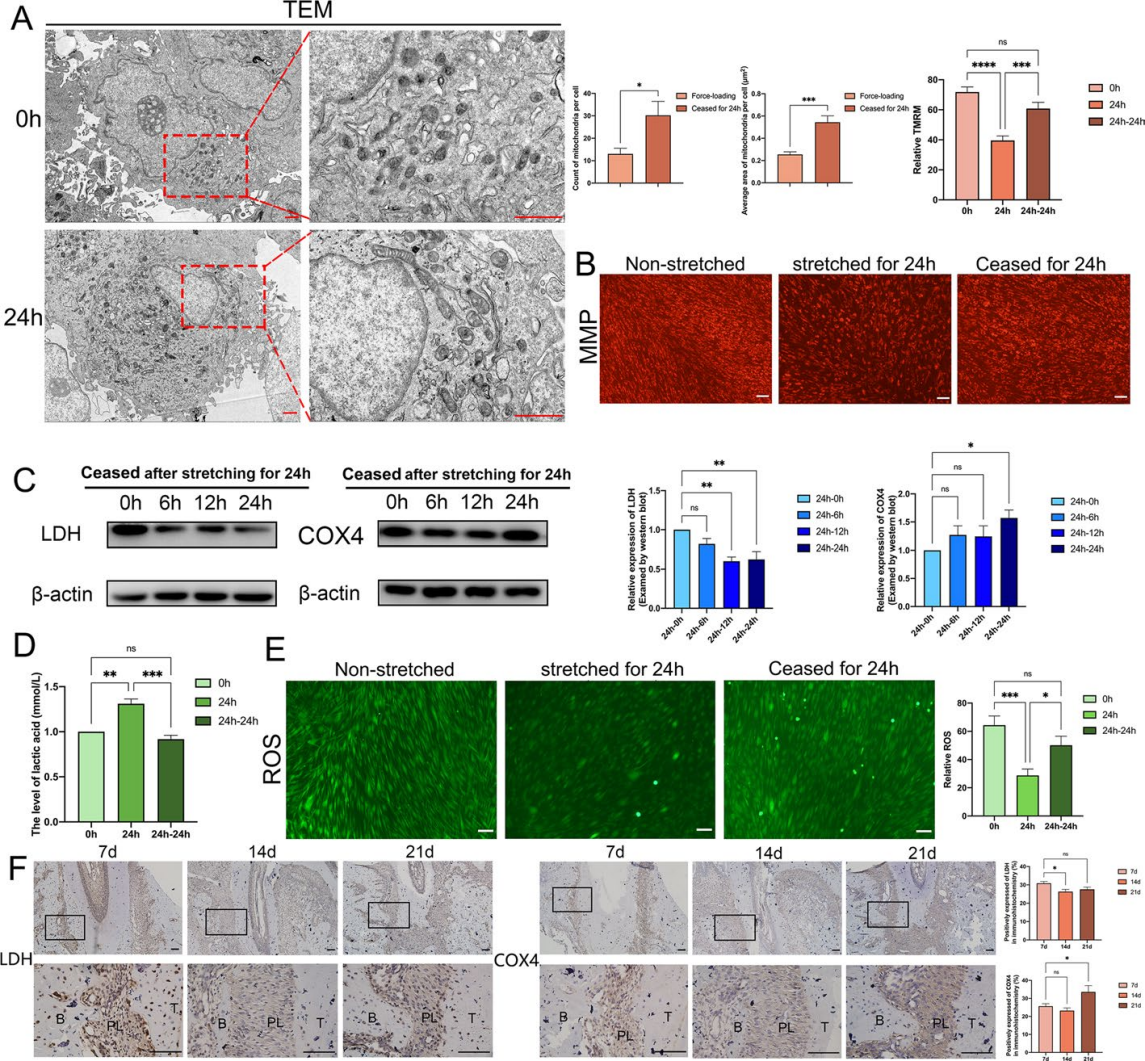
The oxidative pattern altered by mechanical force was restored after the cessation of mechanical force. **A** The morphology of mitochondria in PDLSCs stretched for 24 h and then had the force-loading ceased for 24 h visualized by TEM. Red arrow: mitochondria. Scale bar = 1 μm. **B** The MMP in non-stretched, stretched for 24 h, and rescued for 24 h PDLSCs visualized by TMRM staining. Scale bar = 100 μm. **C** The protein level of LDH and COX4 in PDLSCs rescued for 0 h, 6 h, 12 h, and 24 h after stretching for 24 h. **D** The level of lactic acid in PDLSCs ceased the force-loading for 0 h, 6 h, 12 h, and 24 h after stretching for 24 h. **E** The level of ROS in non-stretched, stretched for 24 h, and rescued for 24 h of PDLSCs. Scale bar = 100 μm. **F** The expression of LDH and COX4 in the periodontal ligament of Wistar rats after 7, 14, and 21 days of tooth movement visualized by immunohistochemical staining. Scale bar = 100 μm. B: alveolar bone, PL: periodontal ligament, T: tooth. *P < 0.05, **P < 0.01, ***P < 0.001, ****P < 0.0001. *ns* no significance

The mechanical force used appropriately can favorably modulate the physiological activities of tissues and cells, whereas the balance of glycolysis and OXPHOS could regulate the physiological characteristics of PSCs. Therefore, whether the regulatory impact caused by mechanical stimulation under physiological settings is reversible means a lot to regulate stem cell fate and maintenance. To test whether the adjustment in the oxidative pattern were recoverable, we ceased the stretching after 24 h of force-loading as a rescue experiment. Compared with the stretching for 24 h group, the number and average size of mitochondria in PDLSCs, which ceased the force-loading for 24 h, increased significantly (Fig. 6A). To measure the restoration of mitochondrial function, we detected MMP in non-stretched, stretched for 24 h, and rescued for 24 h PDLSCs. The level of MMP in PDLSCs rescued for 24 h was significantly elevated compared with the force-loading PDLSCs and showed no significant difference compared with non-stretched PDLSCs (Fig. 6B). Meanwhile, after halting the force loading for 6, 12, and 24 h, we found that the level of LDH lessened significantly, while the expression of COX4 intensified notably after ceasing for 24 h (Fig. 6C). As for metabolites, we ascertained the level of lactic acid and ROS in these three groups. The findings demonstrated that the yield of lactic acid fell back to the initial levels, while the level of ROS was also significantly increased after rescuing for 24 h (Fig. 6D, E). As the validation of simulating the loss of orthodontic force for maintenance stage in vivo, immunohistochemical experiments also confirmed that the expression of LDH significantly reduced at day 14, while the expression of COX4 was significantly boosted at day 21 compared with day 7 of tooth movement (Fig. 6F). Taken together, the oxidative pattern altered by mechanical force could restore after cessation of mechanical stimuli.

## Discussion

As a fundamental part of metabolic characteristics, the glucose oxidation mode should not be only valued from the perspective of energy supply, but also in the aspects of cellular adaptation and fate regulation under external stimulation [24, 25]. Currently, most metabolic-related studies focus on the metabolic reprogramming of cells driven by pathological factors [26], while few studies have been conducted on the potential oxidation mode of stem cells under the stimulation of physiological factors. Our present study mainly concentrated on altering the oxidation mode of PDLSCs under mechanical strains. It was discovered that mechanically-induced mitophagy propelled the anaerobic oxidation of PDLSCs, which could lay a molecular foundation for the maintenance and commitment of potential stem cells by mechanical stimulation.

As an essential nutrient in the metabolic process to meet the need for energy supply, glucose is decomposed by anaerobic oxidation, aerobic oxidation, and pentose phosphate pathway [27] according to specific cellular states and environmental oxygen supply [28, 29]. The best known example of this is the Warburg effect, which shows that cancer cells without mitochondrial defects prefer anaerobic oxidation, ignoring the sufficient oxygen supply [30, 31]. Similar phenomena can be found in somatic cells with high metabolic demands or inflammatory responses [32, 33]. It is noteworthy that stem cells and cancer cells share strong similarities in the metabolic characteristics of glucose utilization, which may be attributed to the rapid proliferation, while anaerobic oxidation provides ATP and necessary metabolic intermediates for the biosynthesis demand [34]. In addition, PSCs show a plastic preferenec for anaerobic oxidation and conservative redox homeostasis, which may realize the control of ROS production and minimize the negative impact of oxidative damage via the maintenance of immature, low-quality mitochondria [11]. OXPHOS is inclined to establish a link with stem cell exhaustion caused by induced lineage commitment, while anaerobic oxidation has far-reaching significance for maintaining stem cell potential [12, 13]. Metabolic reprogramming towards anaerobic oxidation would occur in induced pluripotent stem cells (iPSCs) from reprogrammed somatic cells, which can prove the substantial relevance of anaerobic oxidation to the pluripotency of stem cells [11, 35]. We had assumed that the force-loading would increase aerobic oxidation since mechanical stimulation may cause oxidative stress. Surprisingly, our results point to an augmentation in anaerobic oxidation and a significant reduction in ROS production. We believe that the enhancement of anaerobic oxidation and the fragmentation of mitochondria are consistent with the adaptive protective response of PSCs under external stimulation to achieve stem cell maintenance. As for the alteration of ROS production under mechanical force, we found that the results of other researchers are inconsistent, which may be attributed to the divergent parameters of the force-loading [36, 37]. Our findings may be affected by limitations such as the monotonous experimental condition of a single type of mechanical force. Nevertheless, the consistency of orthodontic tooth movement in vivo and cellular experiments in vitro enhances the persuasiveness of the results. We will also investigate more kind of mechanical forces and their regulation of metabolic profiling in PDLSCs or other periodontal-related cells and tissues and further discuss their mechanisms. Mechanical stimulation, which transcends the physiological range, may cause an upsurge of ROS and cause oxidative stress [38]. Steady and pulsatile mechanical stimulation within the physiological range may induce the rise of ROS for a short time, but the ROS level would decline after the cellular adaptation [39]. These results mentioned above support our findings about the adaptive regulation of anaerobic oxidation of mechanical cues. The link between the mechanical clues of PDLSCs and the regulation of oxidation mode innovatively provides a platform for mechanically-guided stem cell commitment and tissue regeneration.

As the pivotal organelles regulating glucose oxidation, mitochondria exhibit different characteristics between stem cells and differentiated cells [11, 40]. Previous studies have shown that PSCs tend to maintain the mitochondria in a comparatively immature and low-quality state through mitochondrial fusion, fission, and subsequent mitophagy [12, 41]. In the current study, we observed that mechanical stimuli drew forth more fragmentary mitochondrial morphology, increased DRP1, and decreased MFN2. Since the level of mitochondrial fission is determined by the expression level of DRP1 and the mitochondrial recruitment of DRP1 [42], we also evaluated DRP1 recruitment of mitochondria, which supported the hypothesis of augmented fission. Since PDLSCs underwent aggressive mitochondrial fission after being stretched, we speculated that the average number of mitochondria might increase along with their dwindling average size. Unexpectedly, we found that the number of mitochondria also decreased. Mitochondrial fission separated damaged mitochondria with low MMP and conducted the translocation of PINK1 on the outer membrane of depolarized mitochondria to recruit PARKIN to promote PRKN-dependent mitophagy [43]. Additionally, low-quality fragmented mitochondria could be more easily degraded by mitophagy [44]. Therefore, we considered that the partial elimination of fragmentary mitochondria by mitophagy would explain the reduction in the mitochondria number. In this study, the impaired MMP and ascent of mitophagy markers, including MFF and PRKN-dependent pathway-related proteins PINK1 and PARKIN, corroborated the incremental mitophagy in PDLSCs under mechanical force. Our rescue experiment with the mitophagy inhibitor CsA could withdraw the augmentation of anaerobic oxidation, which confirmed the liaison between mitophagy and the amendment of oxidative preference. We noticed that the expression changes of fission-related protein DRP1 were mainly reflected at 24 h after applying mechanical force. In comparison, the mitophagy marker MFF showed significant changes at 6 h after being stretched. Combined with previous reports [21], we also considered that the hysteresis of DRP1 alteration is partly because MFF could be activated by phosphorylation during the prophase of stretching, and it takes time to recruit DRP1 to mitochondria. We studied the cell substructure with alteration of mitochondrial dynamics to explain the mechanism of oxidative preference, providing a molecular foundation for the adaptive response of the organelle under mechanical force.

Since the oxidative profiles of stem cells is crucial for the regulation of stem cell fate related to pluripotency maintenance and differentiation commitment, we wondered whether the alteration in oxidative mode is reversible. According to previous studies, the balance of glycolysis and OXPHOS could regulate the physiological characteristics of PSCs [12, 45]. Generally, OXPHOS could induce lineage commitment and cell proliferation, while anaerobic oxidation tends to realize stem cell quiescence and maintenance [46]. The adjustment of mechanically-induced anaerobic oxidation is consistent with the maintenance of stem cell characteristics and stem cell quiescence [47]. However, as promising “seed cells” for periodontal tissue regeneration, plenty of studies have shown that the proliferation and differentiation of PDLSCs are of great significance for craniofacial tissue regeneration [8, 9]. Therefore, it is necessary to determine whether the application of mechanical force will have an irreversible impact on the differentiated commitment of PDLSCs. In this study, we pinpointed that the oxidative mode of PDLSCs can be reinstated to the baseline after the cessation of mechanical stimulation. From the perspective of oxidative preference, we clarified that mechanical stimulation would not cause irreversible damage to the lineage commitment of PDLSCs for the reversion to aerobic oxidation biased toward the promise of differentiation. These findings are also consistent with the clinical manifestations during orthodontic treatment, which contains the force-induced movement of root position without bone remodeling in the first stage, and PDLSCs-guided osteogenesis and osteoclastogenesis in the second stage of the force-free period [48, 49]. The reversible modification may provide a reasonable explanation of the adaptive response of PSCs receiving external stimulation from the oxidation perspective.

In conclusion, our data provide the first direct evidence of oxidation preference for anaerobic oxidation in PDLSCs under mechanical force and explain its mechanism from the perspective of mechanism-sensitive dynamic equilibrium of pivotal organelles. We will perpetuate the study by follow-up experiments with the different conditions of mechanical stimuli and in-depth discussion on the changes in stem cell characteristics under mechanical forces. Therefore, this study may provide new insights into the adaptive regulation of stem cell fate and the regeneration of homologous craniofacial tissues under mechanical stimuli (Additional file 1).

## Conclusions

In summary, our study confirmed that PDLSCs under mechanical force preferred anaerobic oxidation according to the changes in the levels of oxidation markers and representative metabolites. Afterward, we clarified that the anaerobic preference was induced by mitophagy through the morphology of mitochondria, mitochondrial status, expression of key proteins of mitochondrial dynamics, and functional verification. Our findings lend new mechanistic insight into the regulation and molecular mechanism of mechanical stimulation on the oxidation mode of PDLSCs, which may shed light on the mechanical guidance of stem cell maintenance and commitment, and lay a molecular foundation for periodontal tissue regeneration.

## Supplementary Information

**Additional file 1: **Raw images of western blot used in the figures.

## Data Availability

All data presented in the current study are available from the corresponding author on reasonable request.

## Abbreviations

PDLSCs: Periodontal ligament stem cells
ROS: Reactive oxygen species
MMP: Mitochondrial membrane potential
PSCs: Pluripotency stem cells
COX4: Cytochrome c oxidase subunit 4
ETC: Electron transport chain
LDH: Lactate dehydrogenase
TMRM: Tetramethylrhodamine methyl ester
MFN2: Mitofusin-2
DRP1: Dynamin-related protein 1
MFF: Mitochondrial fission factor
CsA: Cyclosporin A
iPSCs: Induced pluripotent stem cells
