# Supplementary material for: Mechanical force induces mitophagy-mediated anaerobic oxidation in periodontal ligament stem cells
Source: Cell Mol Biol Lett. 2023 Jul 21;28:57. doi: 10.1186/s11658-023-00453-w (PMC10362665; doi:10.1186/s11658-023-00453-w)
Supplement: Supplementary file 1 — Additional file 1: Raw images of western blot used in the figures. [file 11658_2023_453_MOESM1_ESM.docx]

**Raw images of western blot used in the figures:**

***1. The original PVDF membrane and the overall picture of the blots shown in Figure 2B.***

**
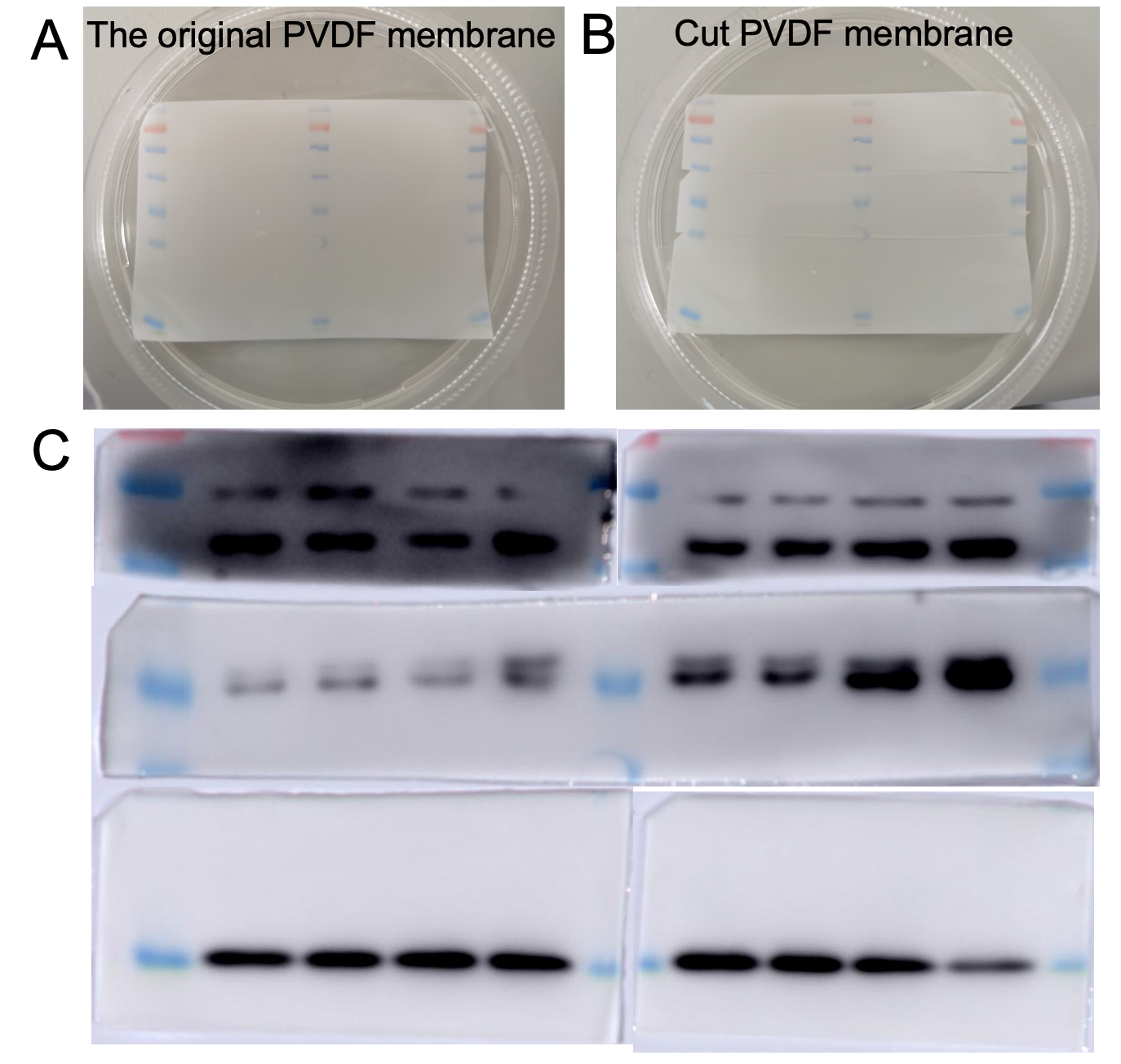
**

***2. The original PVDF membrane and the overall picture of the gels shown in Figure 4C.***


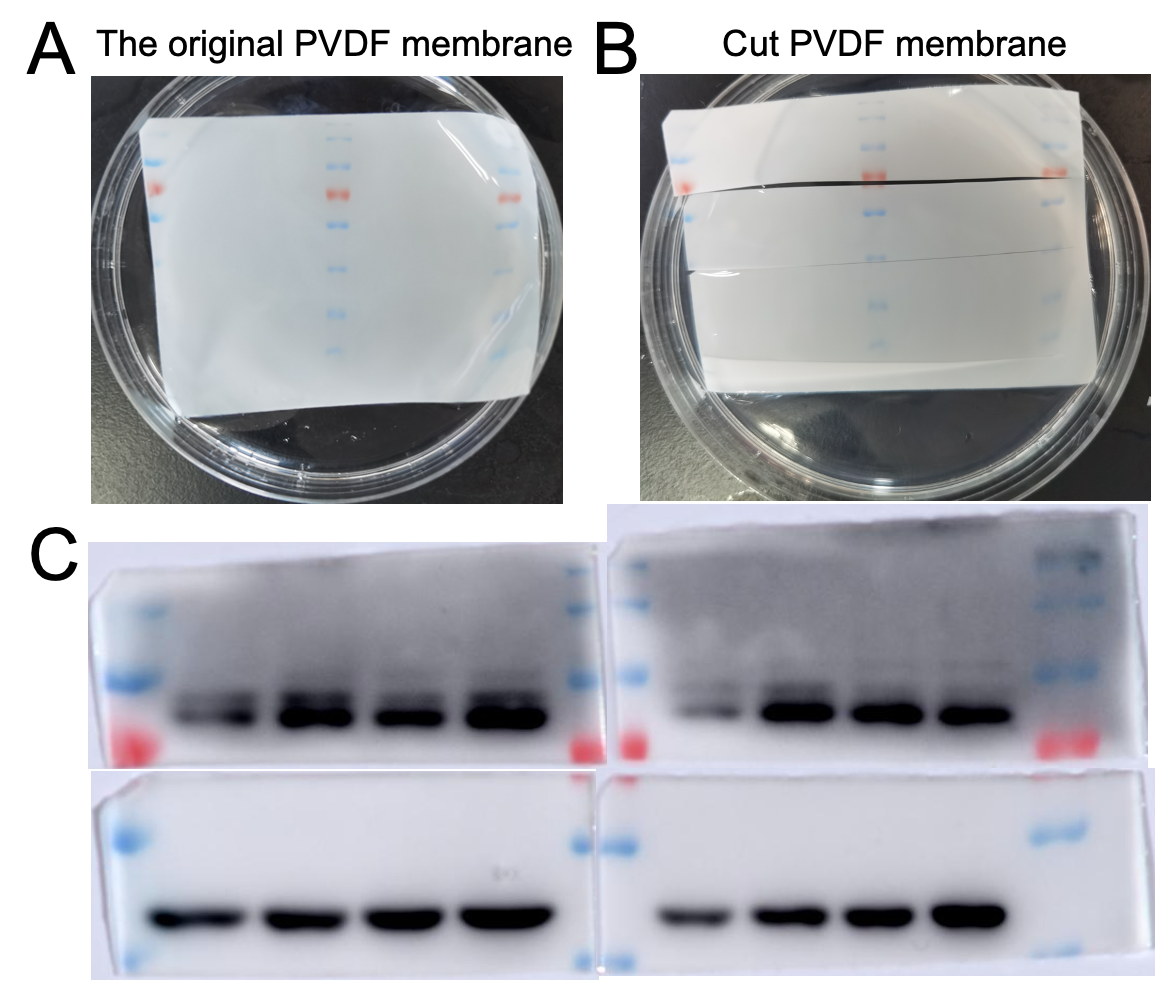

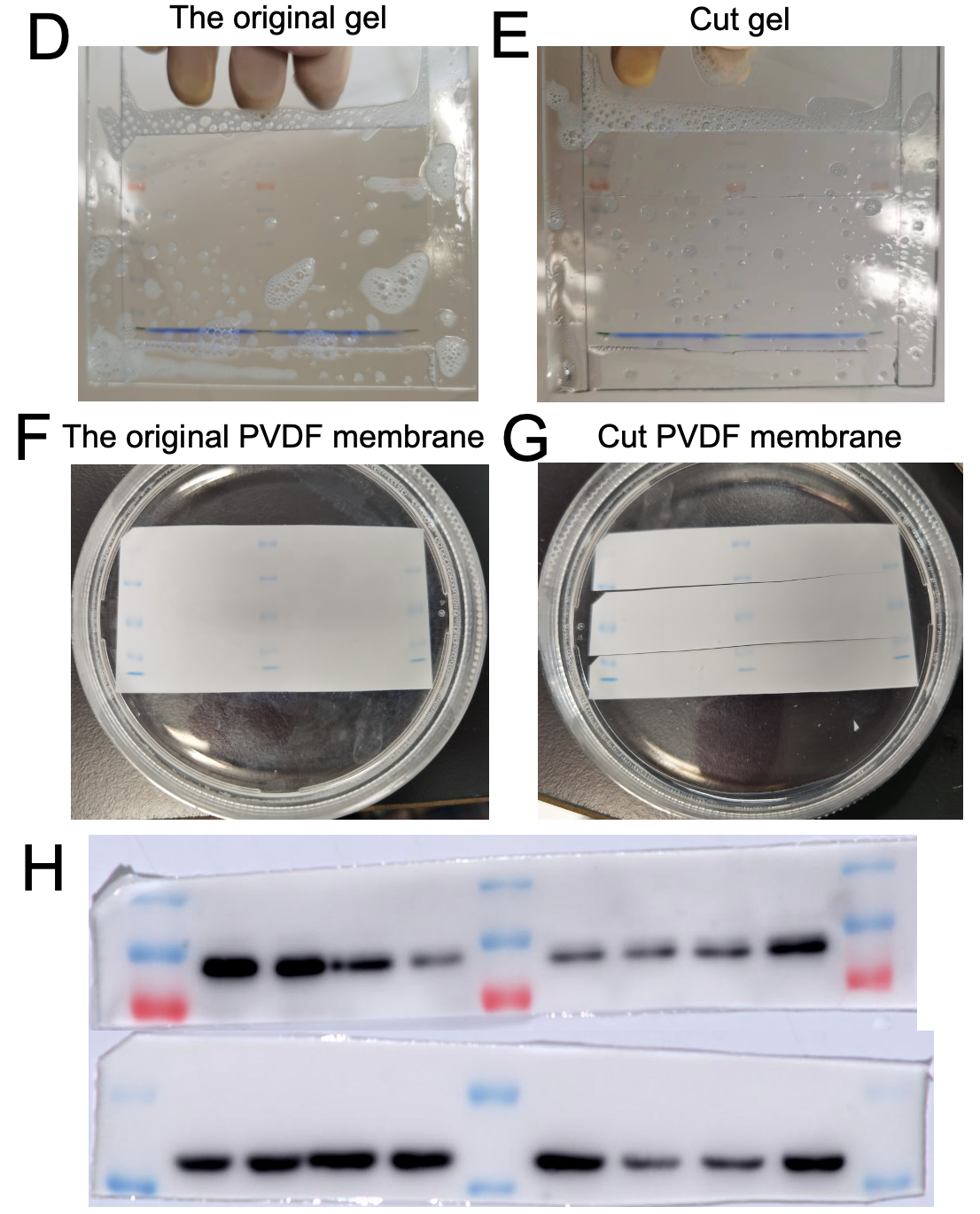


***3. The original PVDF membrane and the overall picture of the gels shown in Figure 5A.***


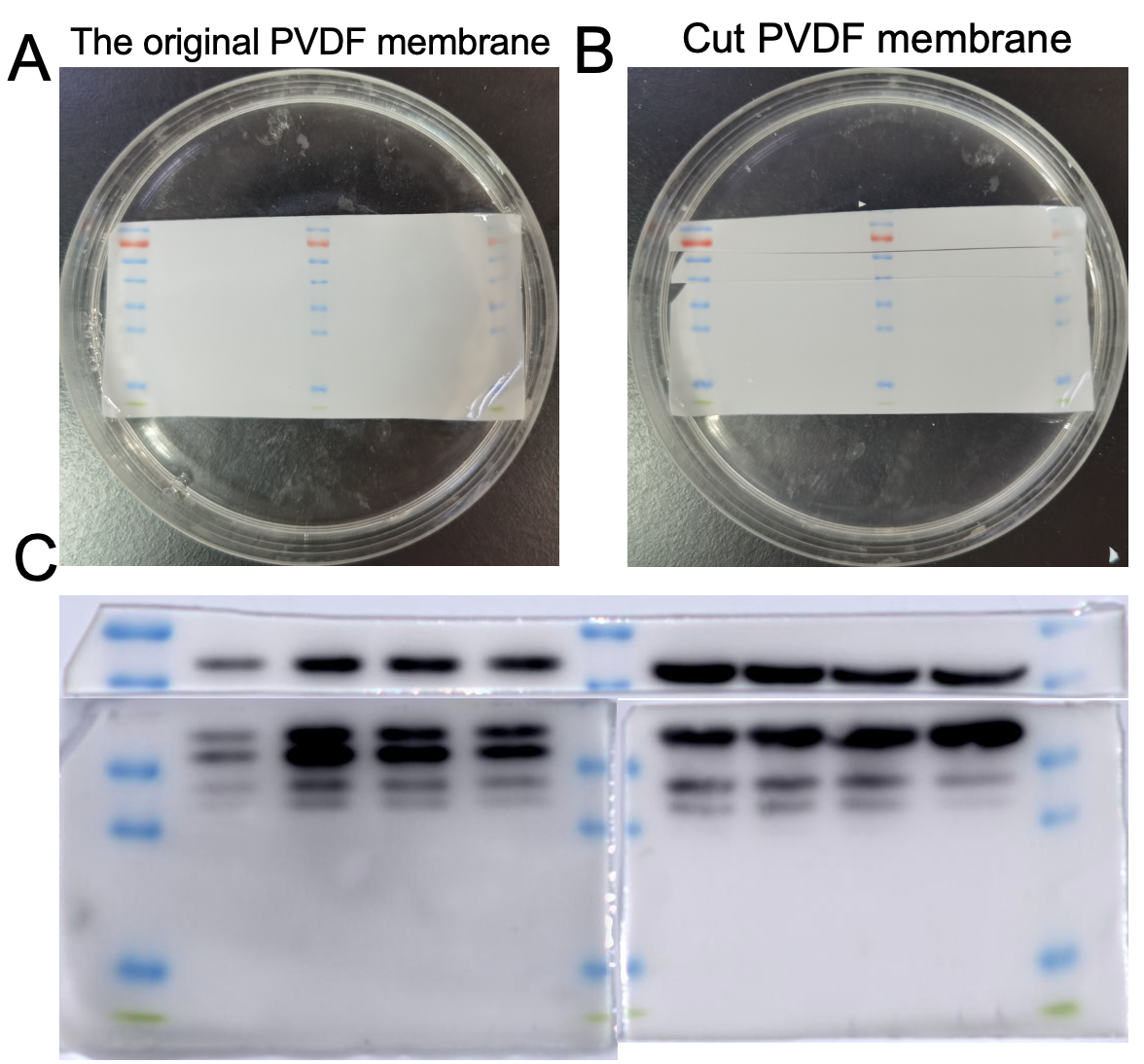

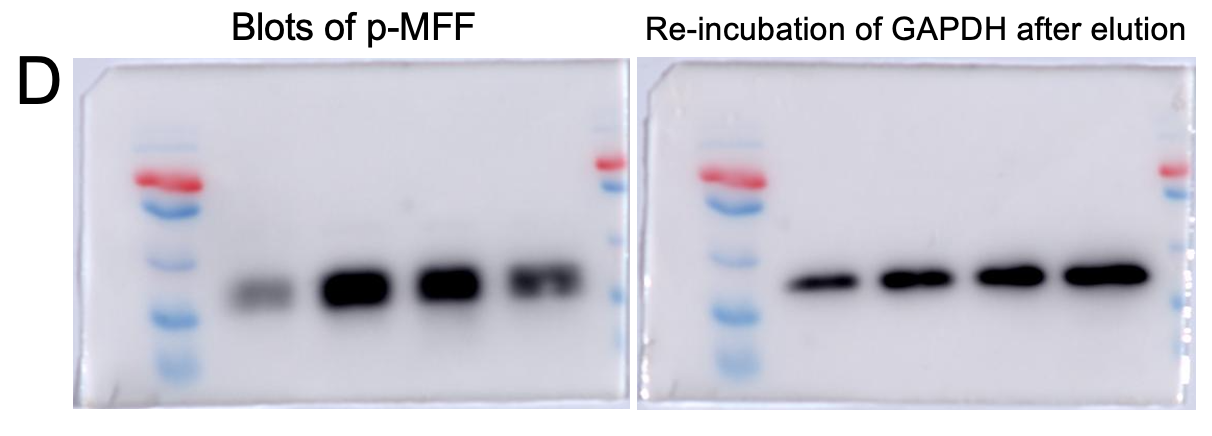


***4. The original PVDF membrane and the overall picture of the gels shown in Figure 5B.***

**
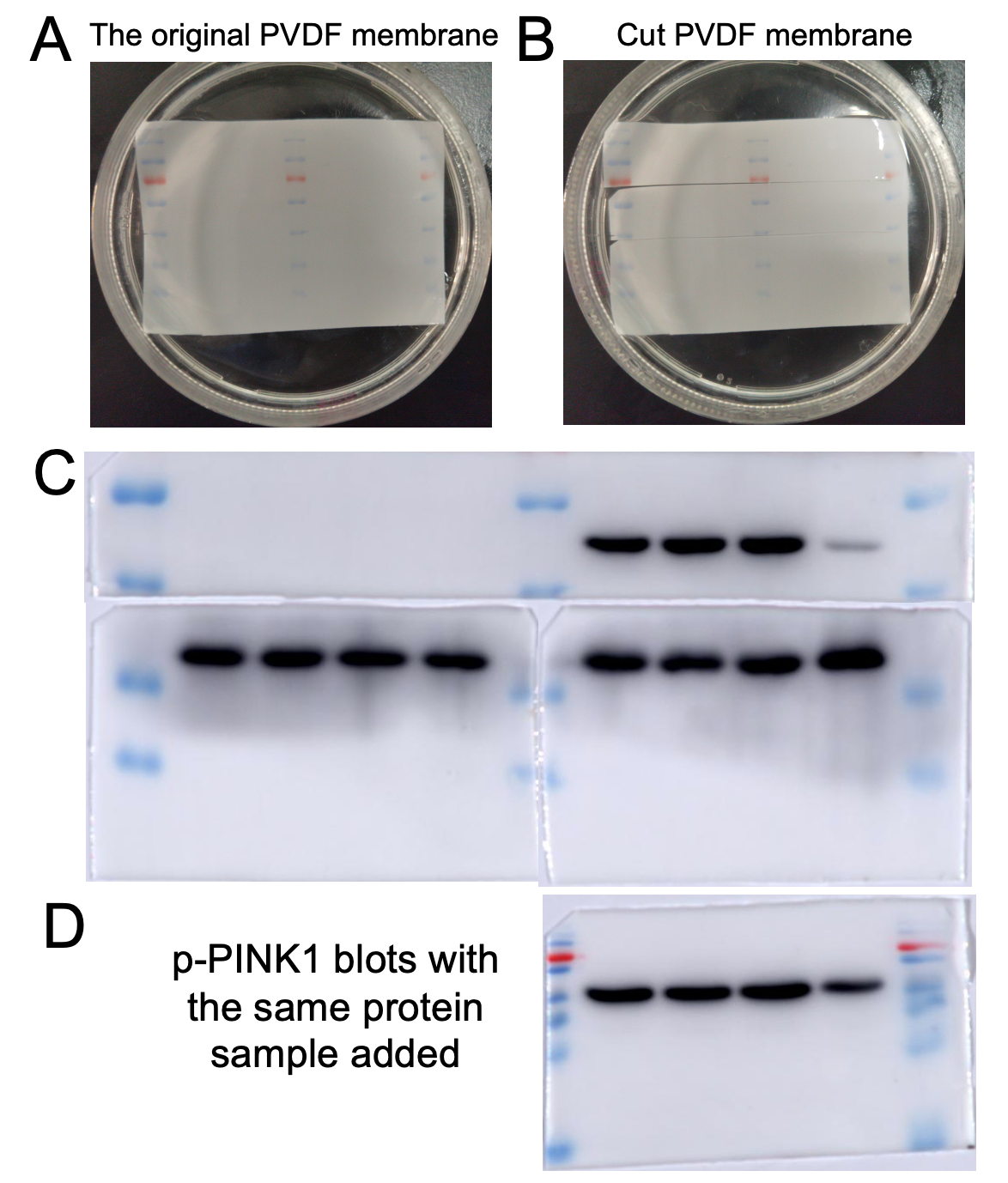
**

***5. The original PVDF membrane and the overall picture of the gels shown in Figure 5C.***

**
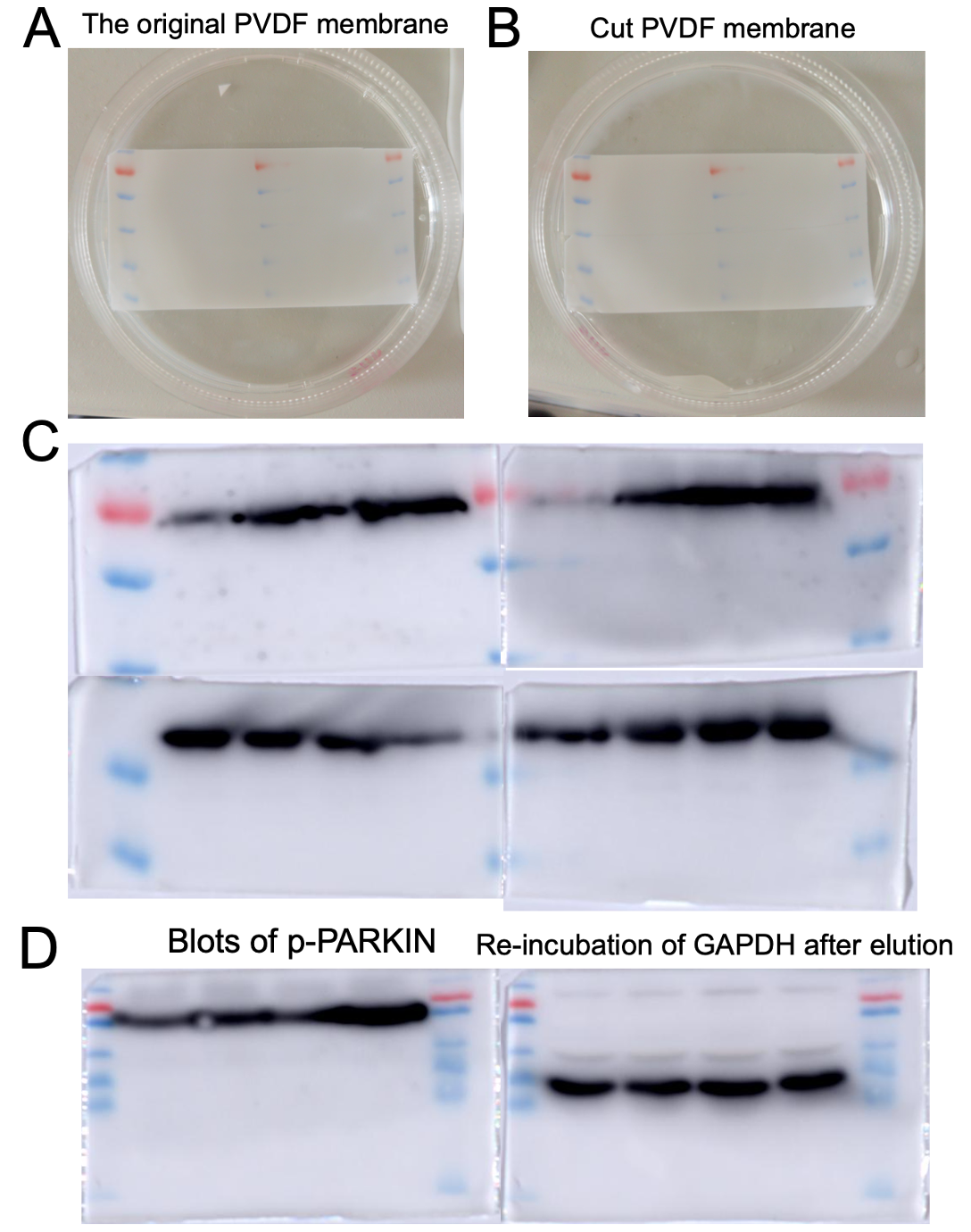
**

***6. The original PVDF membrane and the overall picture of the gels shown in Figure 5E.***

**
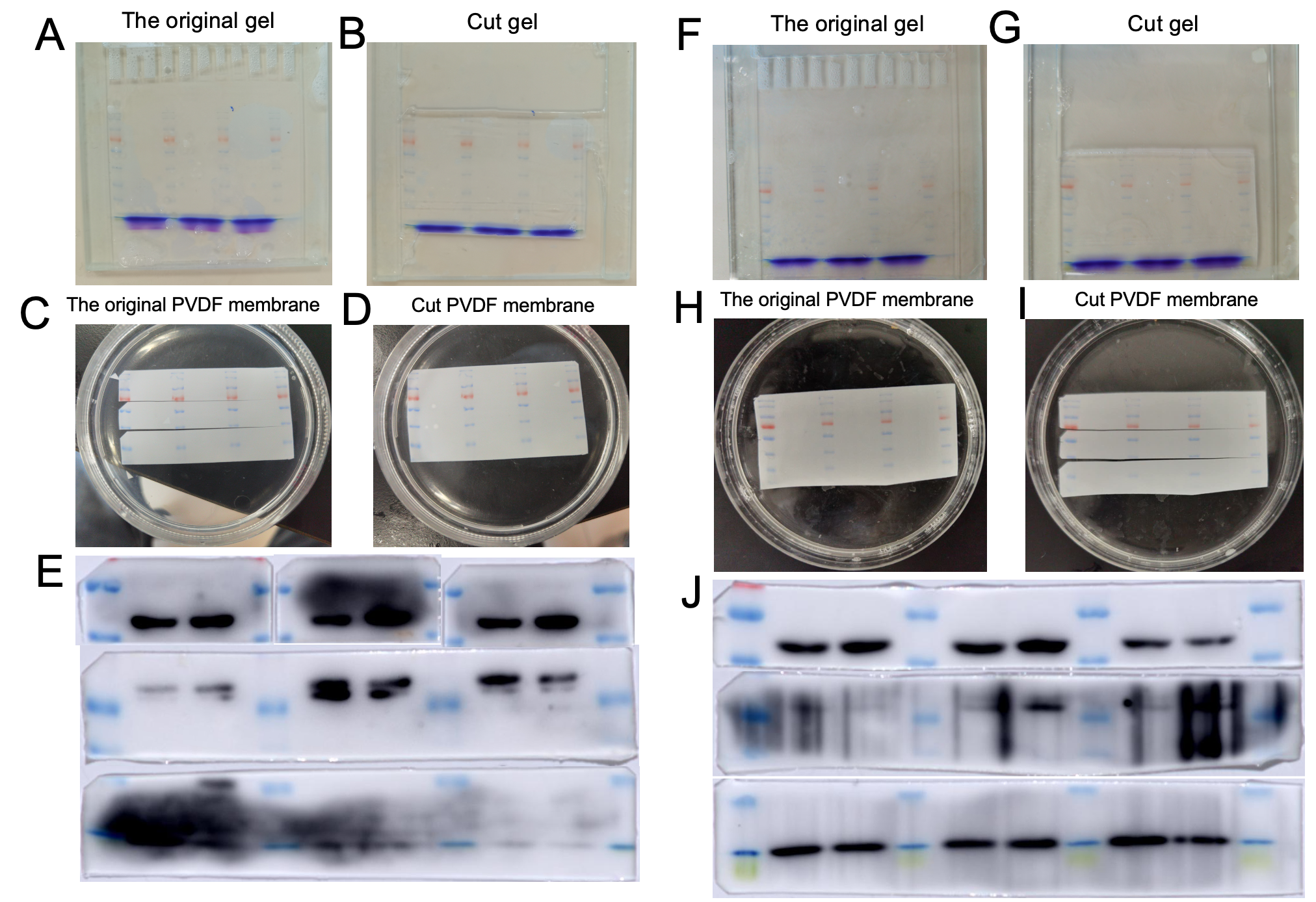
**

***7. The original PVDF membrane and the overall picture of the gels shown in Figure 6A.***

**
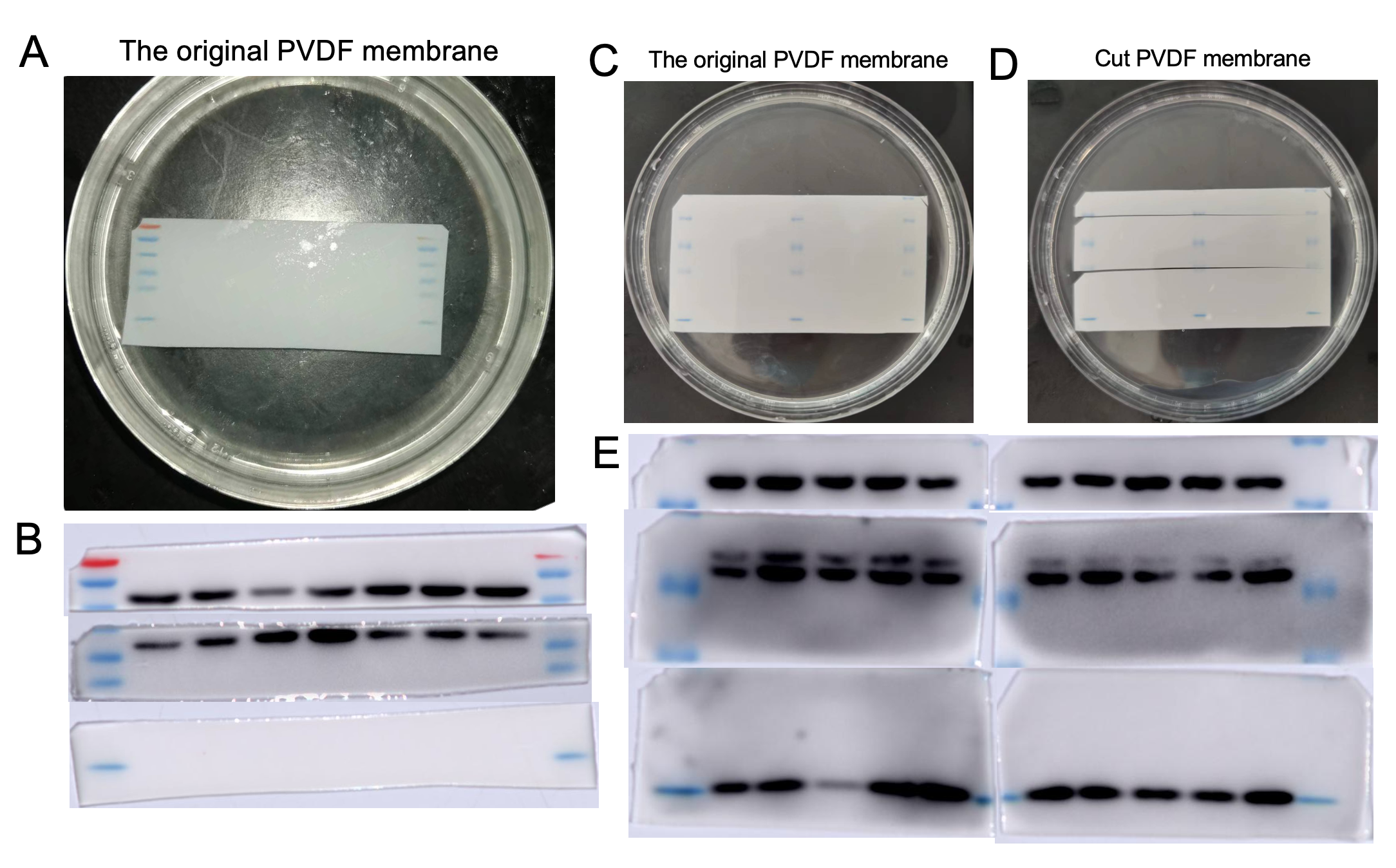
**
